# Characterization and Molecular Insights of a Chromium-Reducing Bacterium *Bacillus tropicus*

**DOI:** 10.3390/microorganisms12122633

**Published:** 2024-12-19

**Authors:** Shanjana Rahman Tuli, Md. Firoz Ali, Tabassum Binte Jamal, Md. Abu Sayem Khan, Nigar Fatima, Irfan Ahmed, Masuma Khatun, Shamima Akhtar Sharmin

**Affiliations:** 1 Environmental Biotechnology Division, National Institute of Biotechnology, Ganakbari, Ashulia, Savar, Dhaka 1349, Bangladeshnigar.fatima1100@gmail.com (N.F.);; 2 Department of Biotechnology and Genetic Engineering, Mawlana Bhashani Science and Technology University, Santosh, Tangail 1902, Bangladesh; 3 Department of Microbiology, University of Dhaka, Dhaka 1000, Bangladesh

**Keywords:** *Bacillus tropicus*, chromium-reducing bacteria, bioremediation, heavy metal tolerant, whole-genome sequencing

## Abstract

Environmental pollution from metal toxicity is a widespread concern. Certain bacteria hold promise for bioremediation via the conversion of toxic chromium compounds into less harmful forms, promoting environmental cleanup. In this study, we report the isolation and detailed characterization of a highly chromium-tolerant bacterium, *Bacillus tropicus* CRB14. The isolate is capable of growing on 5000 mg/L Cr (VI) in an LB (Luria Bertani) agar plate while on 900 mg/L Cr (VI) in LB broth. It shows an 86.57% reduction ability in 96 h of culture. It can also tolerate high levels of As, Cd, Co, Fe, Zn, and Pb. The isolate also shows plant growth-promoting potential as demonstrated by a significant activity of nitrogen fixation, phosphate solubilization, IAA (indole acetic acid), and siderophore production. Whole-genome sequencing revealed that the isolate lacks Cr resistance genes in their plasmids and are located on its chromosome. The presence of the *chrA* gene points towards Cr(VI) transport, while the absence of *ycnD* suggests alternative reduction pathways. The genome harbors features like genomic islands and CRISPR-Cas systems, potentially aiding adaptation and defense. Analysis suggests robust metabolic pathways, potentially involved in Cr detoxification. Notably, genes for siderophore and NRP-metallophore production were identified. Whole-genome sequencing data also provides the basis for molecular validation of various genes. Findings from this study highlight the potential application of *Bacillus tropicus* CRB14 for bioremediation while plant growth promotion can be utilized as an added benefit.

## 1. Introduction

Chromium (Cr) is one of the common pollutants released from various industries, including steel production, electroplating, tanning leather, wood processing, the production of Cr pigments, the manufacture of glass, ceramics, cement, etc. Among these, leather processing industries are one of the highest contributors to Cr pollutants in Bangladesh [1]. In order to turn animal hides into leather, almost all leather tanneries employ chemicals that include toxic elements. The wastewater that these industries release into the environment is one of the main known sources of metal pollution. Approximately 55–70% of the chromium salt that enters the tanning fluid during tanning is fixed in the leather, with the remaining portion ending up in the effluent. The tanning float produces the majority of the chrome effluent, with a sizable part coming from the sammying and re-tanning operations as well. While chromium concentrations in spent liquor range from 2000 to 5000 ppm [2]. International bodies within the EU, such as the Helsinki Commission and the Oslo–Paris Convention, have issued recommendations on chromium discharge levels. The maximum discharge limit to the aquatic environment in the EU is 1 and 5 mg L^−1^ for Cr^VI^ and Cr^total^, respectively [3]. It is conceivable to bring the chromium concentrations in the tannery waste stream down to acceptable levels, but doing so would require significant investment and burden the tanneries with additional operating costs [4].

The toxicity of Cr is significantly dependent on its chemical form. Hexavalent Cr compounds are extremely toxic, mutagenic, and carcinogenic [5]. This is because Cr (VI) is more quickly and actively transported through biological membranes due to its high solubility, bioavailability, and oxidizing capabilities. On the other hand, trivalent Cr (Cr III) is 100 times less hazardous than Cr (VI). Cr (III) in the environment becomes insoluble at neutral or alkaline pH levels and forms a precipitate that is easily removed from aqueous media. A tiny quantity of Cr (III) is even beneficial for many plant species [6,7]. Asthma, a perforated nasal septum, renal failure, dermatitis, hepatitis, and other conditions can make human organs like the skin, liver, kidneys, and respiratory tracts more susceptible to Cr (VI) toxicity. Cr (VI) is more stable in alkaline pH ranges [8,9]. Consequently, the strategy of converting Cr (VI) to Cr (III) in industrial waste is considered to be very useful for reducing immediate toxicity.

To lessen the toxicity of Cr in the environment, research on physical, chemical, and biological processes is being conducted. Many of these techniques are usually costly and inefficient for widespread use. Numerous bacteria possess the ability to convert Cr (VI) into Cr (III), making this bioremediation method more viable, cost-effective, and long-lasting while achieving the highest level of remediation.

Several Cr-resistant bacteria, including *Bacillus* species, have been isolated from chromium-contaminated tannery sites [10,11,12,13]. Bacterial species such as *Chelatococcus daeguensis*, *Sphingopyxis macrogoltabida*, *Pseudomonas alcaliphila*, *Bacillus cereus*, *Klebsiella* sp., *Sporosarcina saromensis* M52, and *Serratia* sp. C8 have been reported as having potential in reducing hexavalent chromium. These bacteria reduce Cr (VI) to Cr (III) in both enzymatic and non-enzymatic reactions [14]. Enzymatic reduction involves specific enzymes, such as reductases, that catalyze the transfer of electrons from a donor molecule to Cr(VI). In bacteria, different chromate reductase enzymes such as ChrR, YieF, NemA, NfsA, and LpDH perform this reaction. These proteins are found either in the cytoplasm (soluble) or in the plasma membrane. Non-enzymatic reduction typically involves redox reactions with cellular components or extracellular electron transfer. Cellular reducing agents, like NADH, NADPH, and glutathione, can donate electrons to Cr(VI), reducing it to Cr(III). Some bacteria can transfer electrons to Cr(VI) through extracellular pathways, such as nanowires or conductive pili, allowing for reduction in Cr(VI) in the environment. However, the specific mechanisms used by bacteria can vary depending on the bacterial species and environmental conditions. 

Investigation at the gene level has revealed various DNA elements in Cr-tolerant bacteria. The *chrR* gene of *Pseudomonas aeruginosa* conferred resistance to chromate. In *Burkholderia mellei*, several chromate resistance genes, namely *chrB*, *chrA*, *chrC*, and *chrF*, have been noted in the chromosomal DNA of which *chrB* and *chrA* genes are essential for establishment of high-level resistance. The genetic studies also showed that *ruvB* gene of *Burkholderia mellei* are related to chromate resistance. In yet another bacterium, *Ralstonia metallidurans*, the chromate resistance determinant *chr2* (comprising genes *chrB2*, *chrA2,* and *chrF2*) was reported to be present on the chromosome. Apart from the genomic DNA, bacterial plasmids also contain genes that are resistant to many toxic metals and metalloids [15].

Interestingly, some of these bacteria exhibit beneficial characteristics toward plants, such as the promotion of plant growth (PGP). It was discovered that the combination of PGP bacteria and particular contaminant-degrading bacteria performed well [16]. PGP has been studied for nitrogen fixation, siderophore production, phosphate solubilization, ammonia, and indole-3-acetic acid synthesis. Therefore, these bacteria have the potential to be used in sustainable agricultural production, particularly in contaminated areas. 

The rate and efficiency of Cr (VI) reduction can be influenced by the initial concentration and environmental factors such as pH, temperature, and the presence of nutrients can affect the activity of chromium-reducing microbes. Moreover, bacteria utilize various metabolic pathways depending on their growth conditions. For instance, *Pseudomonas putida* V1 adopts biosorption, *Bacillus subtilis* employs biotransformation, and *E. coli* utilizes both biosorption and biotransformation processes for reducing Cr (VI) [17,18,19]. Additionally, enzymes in the chromium reduction pathway are typically categorized into two groups. The first group, including the chromium reductase pathway, directly reduces Cr (VI) and its intermediates simultaneously. The second group, involving the nitroreductase pathway, indirectly reduces Cr(VI) through electroconductivity reactions during nitroreduction [20,21,22]. Nonetheless, a considerable knowledge vacuum persists regarding the precise mechanisms behind these actions. Therefore, these mechanisms can be utilized for bioremediation and environmental cleanup if we have a better understanding of how bacteria achieve this Cr reduction and the factors impacting it.

Whole-genome sequencing (WGS) has become an effective approach that allows bacterial genome study and functional difference comparison with the current information. The genomic information anticipates the related pathways which helps in identifying the genes involved in Cr detoxification. This study set out to characterize in detail a bacterial isolate, namely *Bacillus tropicus* CRB14, which was isolated from a local tannery’s effluent and is resistant to several heavy metals, including Cr. We investigated its resistance to Cr (VI), reduction potentials, tolerance to other heavy metals, growth-promoting qualities, growth-promoting effects of pH and temperature, and biofilm formation. To our knowledge, no study has been reported to explore the Cr remediation mechanisms of *B. tropicus*. This study enhances the use of *B. tropicus* CRB14 in the detoxification of heavy metal-contaminated locations by providing a rational basis for understanding chromium resistance and reduction through biochemical and genomic evidence.

## 2. Materials and Methods

### 2.1. Collection and Processing of Sediments

**Figure 1:**
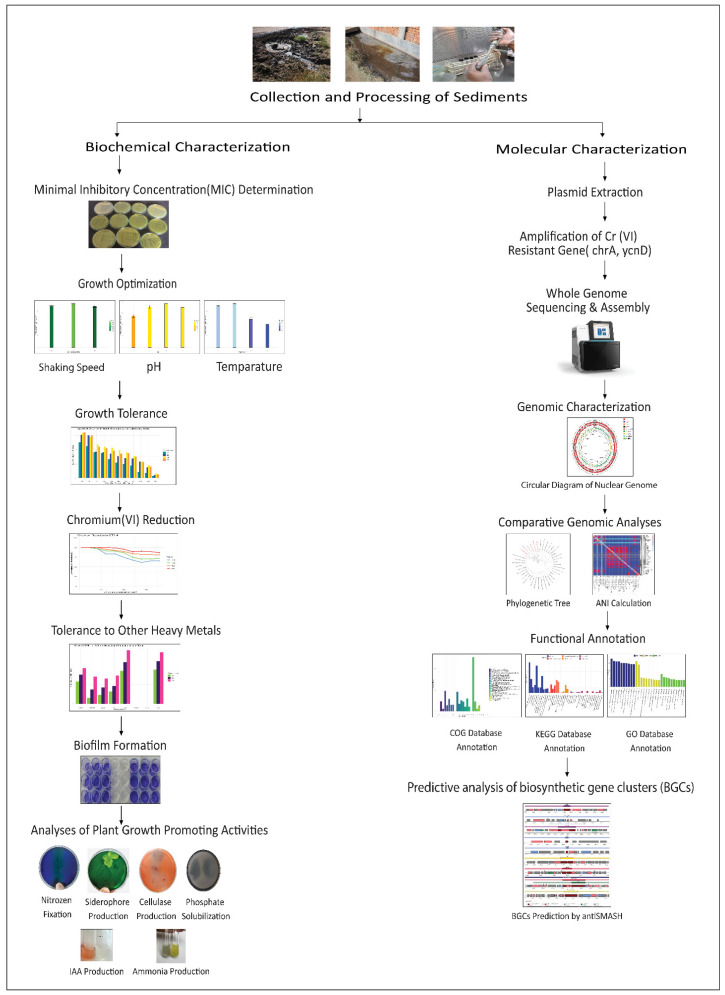
Graphical representation of the complete workflow for characterizing the chromium-reducing bacterium CRB14, starting with bacterial isolation, growth tolerance, and PGP analyses, and progressing through genome sequencing, functional annotation, and comparative analysis.

Tannery sediment was collected from the Hazaribagh tannery area of Dhaka city (Bangladesh) in sterile bottles and immediately transferred to the laboratory. Samples were diluted in normal saline and inoculated (0.1 mL) on Cr(VI)-supplemented (100 mg/L) Luria Bertani (LB) agar plates using the spread plate method. The specified amount of elemental chromium was supplied as potassium dichromate (K_2_Cr_2_O_7_) (Loba Chemie Pvt. Ltd., Mumbai, India). Plates were incubated at 35 °C for 5 days. Useful twenty-five single colonies were selected and subcultured onto fresh LB agar medium and preserved at −80 °C in glycerol stock for further studies. After that, from the stock, we randomly took five isolates for further studies. Figure 1 provides a schematic overview of the entire study.

### 2.2. Minimum Inhibitory Concentration (MIC) Determination

The MIC of Cr (VI) for the isolates was determined on LB agar plates supplemented with filter-sterilized Cr (VI) at different concentrations (1000 mg/L to 6000 mg/L) following the previously described method [23]. After incubation at 35 °C for 5 days, the highest concentration of Cr (VI) which permitted growth and beyond which there was no growth was considered to be the MIC of Cr (VI) for the isolates tested.

### 2.3. Optimization of Physical Factors (pH, Temperature, Shaking Speed) 

To identify the best growth condition, we incubated (Orbital Shaking Incubator, Firstek, New Taipei City, China) the bacteria in LB broth with 100 mg/L Cr (VI) at different pH (6, 7, 8, 9), temperatures (30 °C, 35 °C, 37 °C, 40 °C) and shaking speeds (120 rpm, 150 rpm, 180 rpm).

### 2.4. Growth in Chromium (VI) 

Tolerance to Cr (IV) was performed by growing the isolate in 50 mL LB broth supplemented with 0, 25, 50, 100, 200, 300, 400, 500, 600, 700, 800, 900, 1000 mg/L of elemental Cr (VI) as potassium dichromate for 24 h, 48 h, 72 h, 96 h. Two milliliters aliquot of each sample was withdrawn every 24 h used to evaluate cell growth by measuring optical density at 600 nm, as described by R Baldris et al. (2018) [14]. 

### 2.5. Chromium (VI) Reduction Assay

The reduction in Cr was measured using the diphenylcarbazide (DPC) method. DPC reacts with Cr(VI) to form a purple-colored complex. The intensity of the color is directly proportional to the concentration of Cr(VI). Bacterial cultures were grown in 50 mL LB broth supplemented with 0, 25, 50, 100, 200, 300, 400, 500, 600, 700, 800, 900, 1000 mg/L of Cr (VI). A 1.5 mL aliquot of the original culture (OD 600 = 1.0 ± 0.05) was transferred to each of a series of 50 mL aliquots of LB medium and the mixtures were incubated at 35 °C, pH 8.0 with shaking at 150 rpm for 96 h to investigate the abilities of the isolates to reduce Cr (VI). A 1.5 mL aliquot of each sample was withdrawn every 24 h and the samples were centrifuged (High Speed Microcentrifuge, Hitachi, Japan) at 10,000 rpm for 10 min and supernatants were analyzed for residual Cr(VI) concentration. The reaction mixture was set up in a 1.5 mL tube containing the following: 200 μL sample or standard sodium chromate solution and 400 μL solution (136 μL 3M H_2_SO_4_ and 264 μL 0.25% (*w*/*v*) DPC (Loba Chemie Pvt. Ltd., Mumbai, India). Spectrophotometric measurements (Spectrophotometer U-2910, Hitachi, Japan) were made immediately at 540 nm. The presence of a purple color indicated when hexavalent chromium was present. Cr(VI) concentration in the sample was quantified using a standard plot prepared from K_2_Cr_2_O_7_, as described by R Baldris et al. (2018) [14]. 

### 2.6. Tolerance to Other Heavy Metals

The effect of the various metals (Cd, Hg, Ni, Co, Fe, Zn, and Pb) on the growth of the bacterial isolate was examined in LB broth (5 mL) supplemented with 100 mg/L of selected metals and 50 mg/L Cr (VI). The stock solutions were prepared from the analytical grade salts HgCl_2_, NiCl_2_.2H_2_O, ZnCl_2_, CuCl_2_, NaAsO_2_, PbSO_4_, CdCl_2_, FeSO_4_ (Loba Chemie Pvt. Ltd., Mumbai, India) for Hg (II), Ni (II), Zn (II), Cu(II), As (III), Pb(II), Cd(II), Fe(II) ions, respectively. A corresponding amount of each salt was added to achieve the mentioned elemental concentrations. The tubes were inoculated with 25 μL of the inoculum (OD 600 = 1.0 ± 0.05) and incubated at 35 °C for 72 h. The growth of the bacterial isolate was measured spectrophotometrically at 600 nm [22]. 

### 2.7. Morphological and Biochemical Characterization

The bacterial isolate CRB14 was identified using morphological and biochemical parameters by adopting the standard method [23]. Morphological characterization was performed from pure culture of the isolate grown on LB medium agar at about 35 °C under ambient conditions. The isolate was examined for its size, shape, margin, consistency, elevation, pigmentation, Gram reaction, and cell morphology. A presumptive identification was performed using the following tests: Gram staining, catalase test, oxidase test, urease test, indole test, methyl red-Voges–Proskauer (MR-VP) test, citrate test, starch hydrolysis and nitrate reduction test, carbohydrate utilization test, motility test.

### 2.8. Microtiter Plate Assay (Quantitative Assays for Biofilm Formation)

A crystal violet staining method was employed to examine the biofilm-forming abilities of the isolate with some modifications. Bacteria were first grown overnight in LB broth with or without 25, 50, 100, and 200 mg/mL Cr(VI). Thirty microliters of bacterial culture at phase were inoculated into 96-well polystyrene plates containing 100 μL fresh LB broth and incubated at 35 °C for 24 h. The plates were rinsed three times with deionized water and the adherent bacteria cells were stained with 0.5% crystal violet for 30 min. After being rinsed three times with deionized water, the crystal violet was liberated by 80% ethanol and 20% acetone following a 15 min incubation. The OD values of each well were measured (Microplate Reader, Labtron Equipment Ltd., Camberley, UK) at 492 nm. The tested strains were classified into non-biofilm producer (OD ≤ OD_c_), weak biofilm producer (OD > OD_c_, but ≤ 2 × D_c_), moderate biofilm producer (OD > 2 × OD_c_, but ≤ 4 × OD_c_), and strong biofilm producer (OD > 4 × OD_c_) as described by Baldris et al. (2018) [14].

### 2.9. Analyses of Plant Growth-Promoting Activities 

#### 2.9.1. Production of Indole Acetic Acid (IAA)

The amount of IAA produced by the isolate was determined by following the method described by Gordan and Weber (1951) [24]. Briefly, the bacterial isolates were cultured in LB medium (~10^8^ CFU/mL) and culture was taken afterward to continue the assay. One mL of each culture was transferred into 1.5 mL tubes and centrifuged at 20,000× *g* for 10 min. Then 1 mL supernatant was mixed with 2 mL reagent (Salkowski) for 25 min at room temperature for color formation. The optical density was measured at 530 nm. Using the standard curve for IAA, the amount of IAA produced by each inoculation was calculated.

#### 2.9.2. Phosphate Solubilization Test 

The inorganic phosphate solubilization ability of the bacteria was observed qualitatively by spot inoculation of the bacterial isolate on the NBRIP (National Botanical Research Institute Phosphate) growth medium [25]. The formation of transparent halo zones around the bacterial colonies after 5 days of incubation at 35 °C was considered as an indication of phosphate solubilizing activity. The quantitative estimation of solubilized phosphate was performed using the molybdenum blue method [26]. The bacterial isolates were cultured in NBRIP broth medium (~10^8^ CFU/mL). After 3 days of incubation, each culture was transferred into 1.5 mL tubes and centrifuged at 20,000× *g* for 10 min and phosphorus in the cell free culture supernatant was determined by the above-mentioned method. The intensity of the blue color was read by a spectrophotometer at 882 nm. The quantitative data about the amount of P-solubilized was extrapolated from the standard curve.

#### 2.9.3. Nitrogen Fixation Assay

To determine the nitrogen-fixing capability of the isolate, 100 μL of bacterial inoculum (~10^8^ CFU/mL) was inoculated onto a Petri plate and conical flask containing 50 mL nitrogen-free New Fabian Broth (NFB) media and incubated for 24 h at 35 °C at 150 rpm on a shaker. The amount of fixed atmospheric nitrogen was determined using the Kjeldahl method [27].

#### 2.9.4. Siderophore Production Test 

Detection of siderophore production was performed using Schwyn and Neiland’s universal chrome azurol S (CAS) method [28]. Both qualitative and quantitative methods were used to estimate the siderophore production by the bacterial strain. For both the methods, CAS reagent was prepared as in the above-mentioned method. An uninoculated plate was taken as control. After inoculation, plates were incubated at 35 °C for 3–5 days and observed for the formation of yellow zones around the bacterial colonies. Quantitative estimation of siderophore was performed by taking supernatant of bacterial cultures grown in LB broth medium. After incubation at 35 °C for 48 h, bacterial cultures were centrifuged at 10,000 rpm for 10 min, cell pellets were discarded, and supernatant was used to estimate siderophore. Supernatant (0.5 mL) of the bacterial culture was mixed with 0.5 mL CAS reagent and after 20 min optical density was taken at 630 nm. Siderophore produced by strains was measured in percent siderophore unit (psu) which was calculated according to the method described by Arora and Verma P (2017) [29].

#### 2.9.5. Cellulase Production Test

Pure cultures were screened for cellulase activity by plating on Congo red agar media [30]. Bacterial isolate was streaked on Congo red agar media and incubated at 35 °C for 48 h. The use of Congo red as an indicator for cellulose degradation in an agar medium provides the basis for a rapid and sensitive screening test for cellulolytic bacteria. Colonies showing discoloration of Congo red were taken as positive cellulose-degrading bacterial colonies.

#### 2.9.6. Ammonia Production

To detect ammonia production, bacterial isolate was grown in peptone broth and incubated at 37 °C for 48 h. After incubation, 0.5 mL of Nessler’s reagent was added to the bacterial suspension. The development of brown to yellow color indicated ammonia production [31].

### 2.10. Molecular Characterization

#### 2.10.1. Plasmid Extraction

According to the manufacturer’s instructions, plasmid isolation was carried out using a commercial kit (Pure Plasmid Mini Kit, Cowin Biotech Co., Ltd., Wuxi, China). Final elution product containing any plasmid was visualized with UV light following electrophoresis on 0.8% agarose gel stained with ethidium bromide (0.5 mg/mL).

#### 2.10.2. DNA Extraction and Amplification of Cr (VI) Resistance Gene

Genomic DNA was extracted using a commercial kit (PureLink™ Genomic DNA Mini Kit; Life Technologies Corp., Carlsbad, CA, USA) following the manufacturer’s protocol. The amplification of the chromate reductase gene was carried out by PCR amplification (ProFlex 3 × 32-well PCR system, Thermofisher Scientific, Waltham, MA, USA) using the genomic DNA as template. Primers used are listed in Table 1. PCR reactions were conducted in 25 microlitre aliquots containing 50 ng template DNA, 10 pM of each primer, GoTaq^®^ Green Master Mix, and nuclease-free water. The reaction mixture was subjected to denaturation at 95 °C for 5 min, followed by 35 cycles consisting of denaturation at 95 °C for 30 s, annealing at 54 °C and 53 °C, respectively, for 45 s, and extension at 72 °C for 2 min and a final extension of 72 °C for 10 min. The PCR products were separated on 0.8% agarose gels in TBE buffer stained with ethidium bromide (0.5 mg/mL) and visualized with UV light. 

### 2.11. Whole-Genome Sequencing and Assembly

The DNA library was prepared using standard Illumina DNA prep workflow, which consists of tagmentation of genomic DNA, post-tagmentation cleanup, amplification of tagmented DNA, library cleanup, and library pooling steps. DNA prep insert size was 600 bp. Afterward, whole-genome sequencing was performed in NovaSeq 6000 Illumina platform (2 × 150-bp paired-end reads) using NovaSeq 6000 Reagents (300 cycles) (Illumina, San Diego, CA, USA). The quality of raw sequencing data (.fastq files) was assessed using FastQC v 0.11.8, ensuring reads with high quality (Phred score > 30) [32]. Clean reads were obtained by filtering out adapters and low-quality reads from the processed data using Trimmomatic (v0.39), followed by de novo assembly using SPAdes v4.0.0 [33,34]. 

#### 2.11.1. Genomic Components 

Prokka v1.12 and RAST tools were employed for predicting coding genes, tRNAs, and rRNAs [35,36]. A genomic circular map of the strain was generated using the Proksee web server, integrating GC ratio, GC-skew, and genome sequencing depth data [37]. Genomic islands (GIs) were predicted utilizing the IslandPath- DIOMB GI prediction method of the Island Viewer online tool (version 4.0) [38]. Clustered regularly interspaced short palindromic repeats (CRISPR) loci and antibiotic resistance gene (ARGs) were identified using the CARD Resistance Gene Identifier v1.2.1 tool, utilizing the Proksee web server [39,40].

#### 2.11.2. Genome Identification and Comparison

The 16S ribosomal DNA (rDNA) gene sequence was annotated within the genome, and its homology was assessed by comparing it with 16S rRNA gene sequences from other strains available in the GenBank database [41]. The multiple sequence alignment tool MAFFT (version 7) was utilized for sequence alignment and a phylogenetic tree was constructed from the 16S rDNA using the maximum likelihood method [42]. The resulting tree was then visualized and annotated using the Interactive Tree Of Life (iTOL) version 6.9 [43]. Additionally, the average nucleotide identity (ANI) was calculated using the FASTANI tool on the Galaxy platform (Galaxy Version 1.3) [44]. 

#### 2.11.3. Functional Annotation

An in-depth annotation of predicted coding protein sequences was conducted by comparing them with various databases including Clusters of Orthologous Groups of proteins (COG), Kyoto Encyclopedia of Genes and Genomes (KEGG), Gene Ontology (GO), RefSeq, Pfam, and SwissProt [45,46,47,48,49,50]. COG annotation was performed using eggNOG-mapper v2 to assign putative functions to proteins by identifying orthologous groups across different species based on evolutionary relationships [51,52]. Furthermore, to match predicted genes with the KEGG database, BlastKOALA (Version 3.0) was employed, enabling the identification of genes involved in specific biological pathways via KEGG Orthology (KO) numbers obtained from blast alignment results [53,54]. GO annotation was performed to evaluate the molecular functions of individual gene products, their active cellular components, and the processes they contribute to. GO was annotated using the Gene Ontology web server [46]. 

#### 2.11.4. Prediction of Biosynthetic Gene Clusters (BGCs) 

The antibiotics and secondary metabolite analysis shell (antiSMASH) online version 7.1.0 was used to identify potential biosynthetic gene cluster (BGC) regions, and the resulting job ID was submitted to the biosynthetic gene cluster family (BiG-FAM) database to obtain the corresponding Gene Cluster Family (GCF) classification [55,56]. The antiSMASH analysis was conducted using fasta DNA as input, with a relaxed detection strictness setting.

## 3. Results

### 3.1. Minimal Inhibitory Concentration (MIC) Determination of the Isolates

MIC of the bacterial isolate to Cr (VI) was assessed using the growth response of the strains under 100–6000 mg/L Cr (VI) concentrations for five days. Among the five tested isolates only one isolate, CRB14, was able to grow in up to 5000 mg/L Cr (VI) (Table S1). Therefore, we chose this isolate for further studies.

### 3.2. Effects of Physical Factors (pH, Temperature, Shaking Speed) 

After incubation of the isolates in LB broth with 100 mg/L Cr (VI) at different pH, temperatures, and shaking speeds we found that the bacteria grew well at 35 °C with 150 rpm shaking at pH 8 (Supplementary Figure S1).

### 3.3. Growth Tolerance to Chromium (VI)

**Figure 2:**
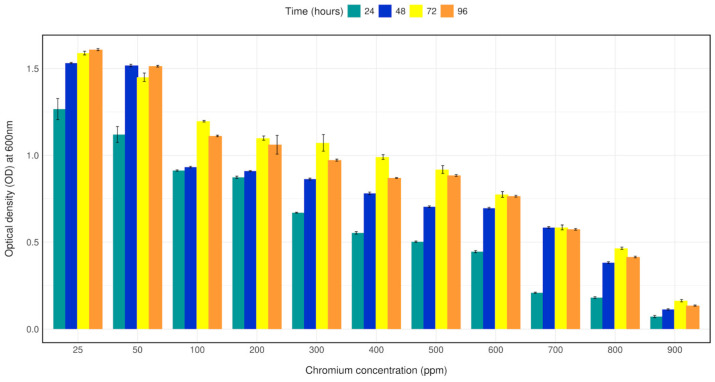
Bar plot depicting the growth of isolate CRB14 at different concentrations of Cr. The cells were cultured on LB broth supplemented with 25, 50, 100 and 200, 300, 400, 500, 600, 700, 800, 900 mg/L Cr (VI). The optical density was measured after incubation for 24 h, 48 h, 72 h, and 96 h at 35 °C.

Bacterial strain CRB14 was exposed to 25–1000 mg/L of Cr (VI) in LB broth for growth tolerance study. The isolate was found to grow up to 900 mg/L of Cr (VI), although growth reduction was observed with increasing concentration of chromium. The bacterial density (OD600) with 25–1000 mg/L Cr (VI) in LB broth was measured after 24 h, 48 h, 72 h, 96 h of culture. Bacterial strain CRB14 showed better growth on Cr (VI) concentration from 25–500 mg/L, a constant decrease in growth of CRB14 was observed at higher concentrations, and no growth was found above 900 mg/L of Cr (VI) (Figure 2).

### 3.4. Chromium (VI) Reduction Profile

**Figure 3:**
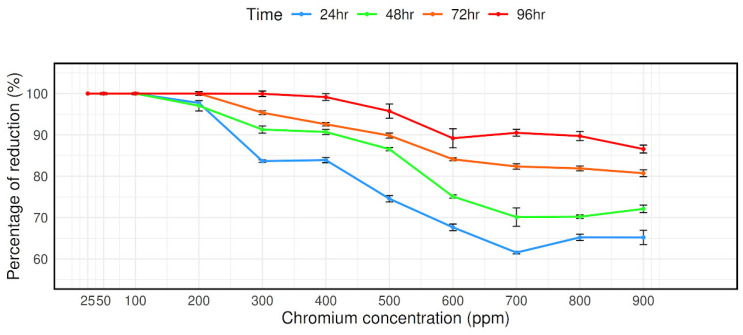
Reduction in Cr (VI) by isolate CRB14. The cells were cultured in Luria Bertani broth supplemented with 0, 25, 50, 100, 200, 300, 400, 500, 600, 700, 800, and 900 mg/L Cr (VI). The Cr (VI) reduction activity was measured after incubation for 24 h, 48 h, 72 h, and 96 h at 35 °C.

The bacterial strain CRB14’s capacity to reduce chromium (VI) was evaluated at concentrations ranging from 25 mg/L to 900 mg/L. As can be observed in Figure 3, at 900 mg/mL Cr (VI) concentrations, it is measured that 65.2%, 72.11%, 80.73%, and 86.57% Cr (VI) reduced after 24 h, 48 h, 72 h, and 96 h of culture, respectively, despite the isolate’s reduction effectiveness steadily decreasing with concentration (Figure 3).

### 3.5. Tolerance to Other Metals

**Figure 4:**
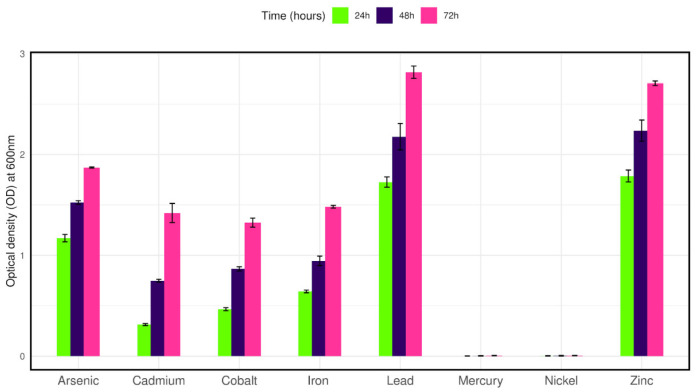
Bar plot representing the growth of isolate CRB14 in the presence of various heavy metals.

To observe the growth tolerance to other heavy metals the isolate was cultured in LB broth containing 100 mg/L of selected metals along with 50 mg/L Cr (VI). The isolate was able to grow in cadmium, cobalt, iron, zinc, lead, and arsenic salts after twenty-four hours of culture. On the other hand, no growth was observed in the presence of nickel and mercury salts after seventy-two hours of culture (Figure 4).

### 3.6. Morphological and Biochemical Properties of CRB14 Isolate

With a high Cr (VI) tolerance and reduction ability, the isolate CRB14 was further subcultured in LB medium for morphological and biochemical characterization. In the LB agar plate, we observed off-white, circular phenotypic characteristics. After Gram staining and microscopic observation, it was found that the isolate is a Gram-positive and rod-shaped bacteria. Biochemical reaction revealed that it is a motile and catalase-positive bacteria. In addition, it could utilize citrate and reduce nitrate. On the contrary, oxidase, urease, indole, methyl red, and Voges–Proskauer tests showed negative results. The bacteria also hydrolyze starch and can ferment glucose and fructose. On the other hand, it failed to ferment lactose and produce H_2_S gas. 

### 3.7. Formation of Biofilm

The isolate CRB14 was tested for biofilm formation cultured at 25, 50, 100, and 200 mg/mL chromium VI. At the elevated level (200 mg/mL of Cr (VI), the isolate produced weak biofilm (OD > OD_c_; (Table S2) according to the criteria of Stepanovic et al. (2007) [57].

### 3.8. Analyses of Plant Growth-Promoting Abilities

We observed the plant growth-promoting properties in CRB14. This isolate fixed atmospheric N_2_ as it could grow in a nitrogen-free New Fabian Broth (Nfb) medium. The amount of nitrogen fixed by the isolate was 10.34 μg/mL. It was also able to solubilize insoluble phosphate in NBRIP media indicating its potential to promote the growth of plants under phosphate-limited conditions. In quantitative analysis, the isolate could solubilize phosphate 1.05 μg/mL. The isolate in its respective culture of ~10^8^ CFU/mL was screened for their ability to produce IAA. The IAA production was found to be 28.165 μg/mL. In continuation of addressing PGP activities, this study characterized the siderophore-producing ability of the test isolate. The siderophore-producing ability was scored qualitatively by the formation of orange zones produced around the bacterial growth. In a quantitative approach, it was observed that it can produce 49.02 psu (percent siderophore unit). Moreover, it degraded cellulose and showed the ability to produce ammonia (Supplementary Figure S2).

### 3.9. Molecular Profile

#### Cr-Associated Genes

The genetic determinants of resistance to heavy metals are often found in plasmids and transposons [58]. Therefore, we tested if there were any detectable plasmids present. Upon plasmid extraction and gel electrophoresis, isolate CRB14 showed no plasmid. This finding suggests that tolerance to Cr by CRB14 is possibly mediated by chromosomal genes.

To investigate whether CRB14 isolate possesses some known heavy metal tolerance genes, PCR amplification of specific chromium tolerant genes *chrA* (chromate transport protein), and *ycnD* (FMN reductase) was conducted using genomic DNA. Analysis of the PCR product showed that the isolate produced a band of 268 bp, the expected size for the chrA gene according to Patra et al. (2010), indicating that CRB14 contains chromium-resistance gene in its chromosome (Supplementary Figure S3) [59]. However, no band was detected for the *ycnD* gene. The presence of the *chrA* gene was further confirmed by sequencing the PCR product.

### 3.10. Genomic Characterization of Isolate CRB14

**Figure 5:**
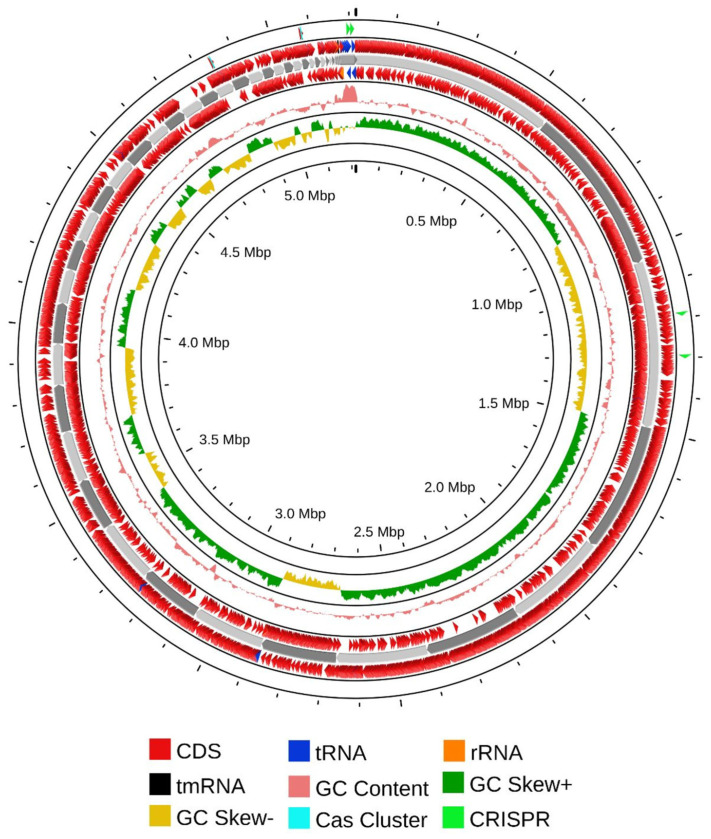
Nuclear genome circle diagram of CRB14. From outside to inside, coding genes (positive-sense strand), coding genes (negative-sense strand), tRNA (blue) and rRNA (orange), tmRNA (black), CRISPR (green), Cas cluster (cyan), and GC ratio and GC-skew.

The aforementioned experimental research was further validated through comprehensive genome sequencing and analyses. Sequencing of the genomic DNA revealed that the complete genome of CRB14 was assembled into 344 contigs, totaling 5,217,143 bp with an average GC content of 35.3%. A total of 5255 genes were predicted, including 2043 hypothetical proteins, 3 rRNA, 37 tRNA genes, and 1 tmRNA gene. Additional genome features are presented in the Table S3. The circular view of the genome from PROKSEE online software (v1.0.0a6) is presented in Figure 5 highlighting the GC distribution, and GC-skew of the genome. The genome data for CRB14 has been submitted to NCBI under Biosample accession number SAMN41810974 and BioProject ID PRJNA1123424.

### 3.11. Genomic Islands and CRISPR Prediction

Genomic islands (GIs) are vital for microbial genome evolution as they harbor diverse genes that enhance adaptability to the surrounding environment. These regions influence key traits contributing to pathogenesis, antibiotic resistance, metabolism, etc. Thus, the prediction of GIs has become increasingly essential in microbial genome analysis 34 putative GIs were found in CRB14 and the size of GIs ranged from 5.7 to 38.9 kb [60]. CRISPR guide RNAs can be tailored to target pathogen-specific virulence and chromosomal genes, allowing for the repurposing of CRISPR-Cas systems to combat bacteria rather than solely defending against external threats. Moreover, CRISPR-Cas9 technology has the potential to generate antibiotics that specifically target antimicrobial-resistant pathogens by precisely targeting their genetic sequences. Therefore, detecting CRISPR elements within microbial genomes is crucial for gaining insight into the dynamics of microbial communities [61]. Four CRISPRs, four cas genes, and two cas clusters were involved in CRB14. 

### 3.12. Heavy Metals and Antibiotic Resistance Genes

Biochemical characterization of *Bacillus tropicus* CRB14 demonstrated its ability to grow in the presence of various heavy metals, including chromium, cadmium, cobalt, iron, zinc, lead, and arsenic. To further support these findings, the annotated genome of CRB14 was analyzed for the presence of heavy metal tolerance genes. The results revealed abundant genes associated with resistance to chromium, cadmium, cobalt, and zinc (Table 2). These genomic findings align closely with the biochemical data, reinforcing CRB14’s capacity to tolerate and survive in environments contaminated with heavy metals.

Non-antibiotic compounds, such as antibacterial biocides and metals, frequently lead to the selection of bacterial strains that can also resist antibiotics. This dual resistance arises through two primary mechanisms: co-resistance, where resistance genes for both antibiotics and metals/biocides are located within the same cell, and cross-resistance, where a single mechanism (such as an efflux pump) provides resistance against both antibiotics and metals/biocides [62]. In light of this, the genome of CRB14 was evaluated for its resistance to antibiotics. The findings indicated that it was sensitive to several antibiotics, including vancomycin, teicoplanin, fosfomycin, tetracycline, and doxycycline, among others. In addition to the chromium resistance gene, it also harbors genes resistant to cobalt, zinc, cadmium, and copper. A detailed list of genes related to antibiotics and the associated resistance mechanisms is provided in Table S4.

### 3.13. Genome Comparison

**Figure 6:**
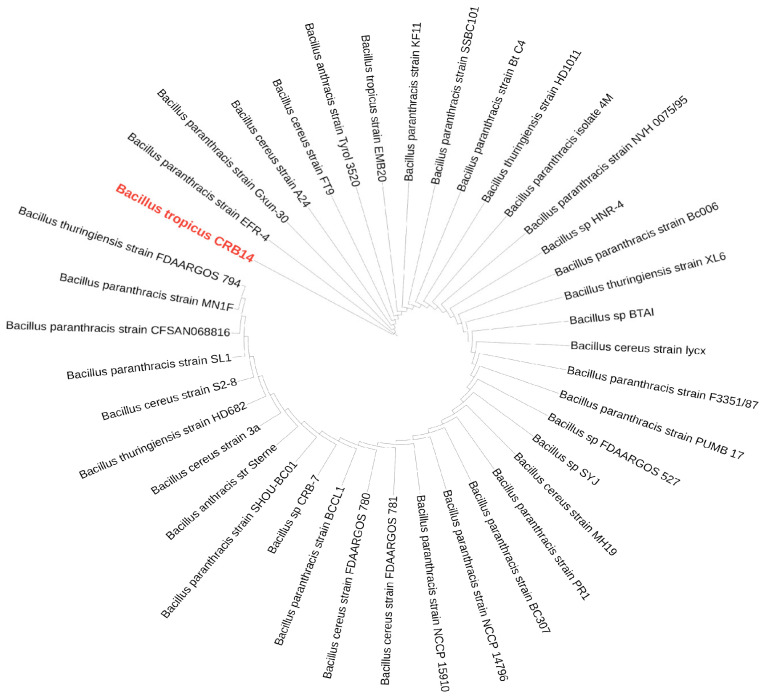
A maximum-likelihood phylogenetic tree based on 16S rRNA gene sequences of *Bacillus tropicus* CRB14 and other closely related strains. The isolate of interest is highlighted in red.

In the BLAST search of the NCBI database using the 16S rDNA sequence of CRB14, numerous strains were identified. From these, 39 strains were selected for further analysis, as they exhibited 100% sequence similarity (E-value 0.00) to CRB14, meeting the species delineation threshold of 98.65% 16S rDNA sequence similarity [63]. The phylogenetic relationship of CRB14 depicted in Figure 6, shows its connection with closely related strains from GenBank. The genomic similarity between CRB14 and other strains was further assessed, enhancing the accuracy and resolution of the phylogenetic signals to demarcate bacterial species [64]. CRB14 exhibited an ANI value of 99.51% with *Bacillus tropicus* strain EMB20, leading to the identification of CRB14 as *Bacillus tropicus* (Supplementary Figure S4).

### 3.14. Functional Gene Annotation

#### 3.14.1. COG Database Annotation

**Figure 7:**
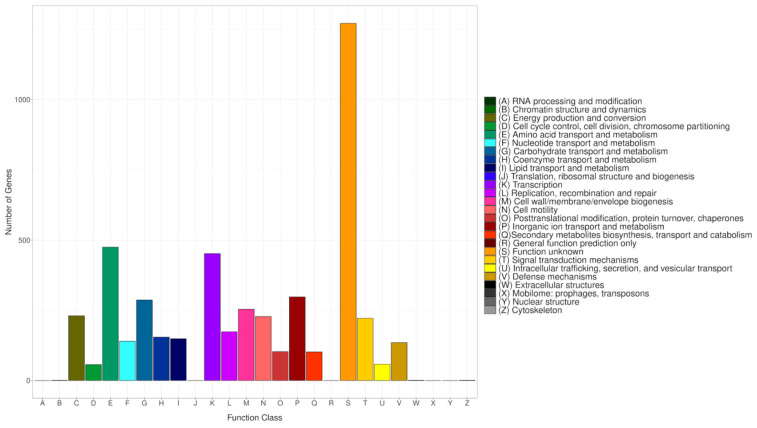
COG classifications of the genome. Each bar corresponds to a specific classification, highlighting these proteins’ diverse roles in metabolism and physiological processes. The abscissa represents the various COG categories, while the ordinate shows the number of genes assigned to each category.

The COG database categorizes proteins from fully sequenced genomes based on the principle of orthology [65]. To align all predicted CDS sequences of CRB14, the COG database was employed, utilizing the genome for functional and comparative analysis. Altogether 4794 protein-coding sequences were successfully annotated into COG (Figure 7), categorized into 26 groups ranging from A to Z. A substantial number of genes were classified into the categories of “Amino acid transport and metabolism” (475), “Transcription” (452), “Inorganic ion transport and metabolism” (298), and “Carbohydrate transport and metabolism” (287). Meanwhile, genes associated with “Signal transduction mechanisms” (222), “DNA replication, recombination, and repair” (174), and “Secondary metabolites biosynthesis, transport, and catabolism” (102) were also present in notable proportions. The number of genes in the S (Function unknown) group is enormous. However, the current database failed to clarify their biological functions, indicating that many unknown functions are worthy of further elucidation. 

#### 3.14.2. KEGG Database Annotation

**Figure 8:**
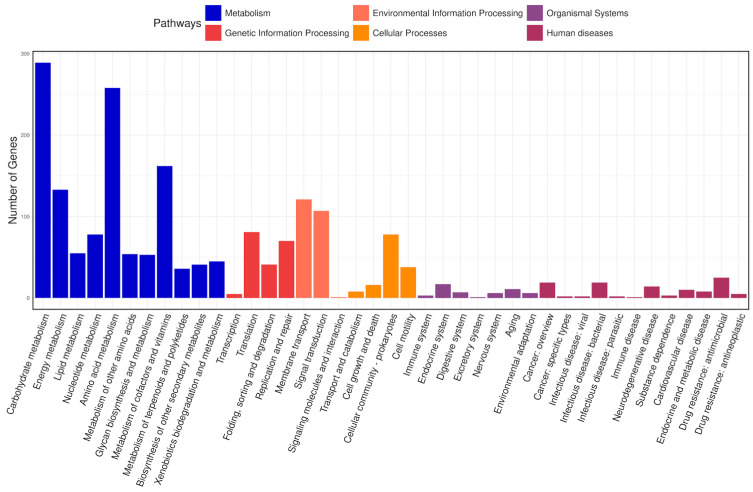
KEGG classification of the predicted coding sequences. The x-axis denotes the various pathways, and the y-axis indicates the number of genes assigned to each pathway. The bars are color coded according to the six major pathway classes, indicating that the majority of genes are involved in metabolism.

The KEGG database connects molecular functions of genes and proteins to ortholog groups, aiming to relate gene sets in the genome to the higher-level functions of cells and organisms [66]. In total, 2686 genes were classified into 41 pathways in the KEGG database and arranged into eight fundamental classes, each comprising several subclasses (Figure 8). Most genes fell into the following categories: under “metabolism”, there were 289 genes involved in carbohydrate metabolism, 258 in amino acid metabolism, 162 in the metabolism of cofactors and vitamins, and 133 in energy metabolism; under “environmental information processing”, 121 genes were related to membrane transport and 107 to signal transduction; and under “genetic information processing”, 81 genes were associated with translation and 70 with replication and repair. 

#### 3.14.3. GO Database Annotation

**Figure 9:**
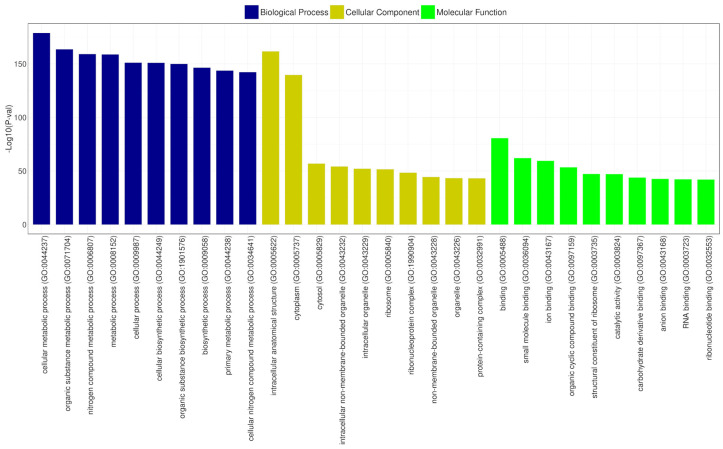
GO functional classification of CRB14. The x-axis shows the GO categories, and the y-axis represents the −log10 (*p*-value) for the top 10 terms in biological process (blue), cellular component (yellow), and molecular function (green).

Gene Ontology (GO) is a structured vocabulary project designed to describe the functions of gene products across all organisms. It categorizes molecular and cellular biology into three domains: molecular function, cellular component, and biological process, each with its subdomains [67]. According to the GO functional annotation results for CRB14, the cellular components domain was predominantly represented by genes related to intracellular anatomical structures and the cytoplasm. Similarly, the biological process category revealed that most genes were associated with cellular metabolic processes and organic substance metabolism. In the molecular function domain, the most prevalent functions were binding, ribosomal structural components, and catalytic activities. Figure 9 depicts the most significant subdomains within each major domain of the GO database.

### 3.15. Predictive Analysis of Biosynthetic Gene Clusters (BGCs)

**Figure 10:**
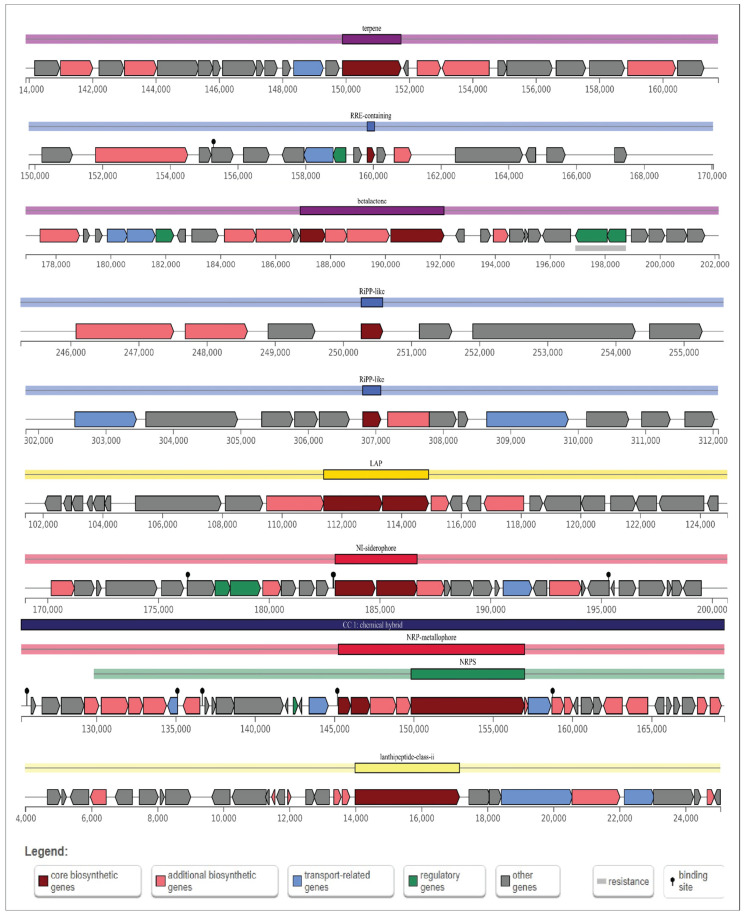
Schematic diagram of nine secondary metabolite biosynthetic gene clusters in *B. tropicus* CRB14. Potential secondary metabolite biosynthetic gene clusters were predicted using antiSMASH. Color-coded blocks indicate different gene functions: dark red for core biosynthetic genes, light red for additional biosynthetic genes, blue for transport-related genes, green for regulatory genes, and gray for other genes.

AntiSMASH predicted a total of nine gene clusters for secondary metabolite synthesis: Tarpene, RRE-containing, Betalactone, RiPP-like, Linear azol(in)e-containing peptides (LAP), NRPS-independent, IucA/IucC-like siderophores (NI-siderophore), non-ribosomal peptide metallophores (NRP-metallophore), and Class II lanthipeptides like mutacin II (U40620). There are three strictness levels for cluster detection: strict, relaxed, and loose. RRE-containing and RiPP-like clusters are in the relaxed level and the rest of them in the strict level. All these groups of genes included genes involved in additional biosynthetic, core biosynthetic, transport-related, and regulatory functions, as well as other genes (Figure 10), of which siderophore and NRP-metallophore have been widely studied. These are the most effective ways for microorganisms to take up iron from iron-poor environments: Bacteria containing siderophores use specific ATP-dependent membrane-associated transporters to deliver the Fe (III)-siderophore complex to the cell. 

Inside the cell, Fe(III) is enzymatically reduced to soluble and solid-phase Fe(II), which quickly transfers its electron to Cr (VI), becoming the reductant for Cr (VI) [68]. The biosynthetic gene cluster family (BiG-FAM) database is used to show the summary of all best BGC-to-GCF pairings with a distance lower than 900 (original threshold value) depicting a good match to at least one GCF that belongs to the genus *Bacillus* in the database and the completeness of the gene (Table S5).

## 4. Discussion

Widening the understanding of heavy metal detoxifying bacteria is crucial for their utilization for bioremediation purposes. Moreover, evidence-based information potentially acts as a source of genetic engineering. This study investigated a promising bacterial isolate, CRB14, showcasing its excellent tolerance and reduction capabilities towards chromium (VI), a toxic heavy metal contaminant. 

The isolate demonstrated an exceptional tolerance to chromium (VI), thriving in concentrations as high as 5000 mg/L in agar and 900 mg/L in broth. Chromate reduction to chromium produces radicals, which make this metal very toxic [69]. In solid medium, the bacterial cells might reduce chromium (VI) adjacent to cells and create a less toxic environment surrounding them. On the other hand, in shaking broth culture, chromium is more soluble and consistently homogenous, more readily available, and therefore more inhibitory. In several studies, a higher MIC for chromium was also observed in agar plates compared to the broth culture [70,71].

Nevertheless, such tolerance is particularly noteworthy as chromium (VI) is highly toxic to most microorganisms. Furthermore, CRB14 exhibited significant chromium (VI) reduction ability, achieving over 86% reduction within 96 h at higher concentrations. This reduction capability is crucial for bioremediation strategies as it transforms chromium (VI) into its less toxic form, chromium (III). Previously reported, *Bacillus cereus* isolate PGBw4 mitigated toxic effects of Cr (VI) more efficiently from 100 mg/L to 500 mg/L in LB broth and *Bacillus amyloliquefaciens* has tolerance to Cr(VI) up to 860 mg/L in LB broth [72,73]. The Gram-negative bacteria *Acinetobacter haemolyticus* exhibited high performance in Cr(VI) reduction at low concentrations of 10–30 mg/L, unlike the incomplete reduction performance at a higher concentration of 70–100 mg/mL [74]. In addition, it is found that presumptively identified *Bacillus* sp. can tolerate 7700 mg/L Cr (VI) in LB agar media and can reduce 1000 mg/L Cr (VI) in LB broth media. However, their reduction efficiency is only 44.33% in 500 mg/L Cr (VI) in LB broth media [75]. It is noteworthy that Cr reduction did not completely relate to its tolerance level. Hence, it would be wise to select isolates for remediation purposes based on their Cr(VI) reduction capability rather than their high tolerance to Cr(VI) [76].

Besides chromium, CRB14 demonstrated resistance to an array of heavy metals, including cadmium, cobalt, iron, zinc, lead, and arsenic, highlighting its potential for broader application in environments contaminated with multiple metals. Notably, the isolate failed to grow in the presence of nickel and mercury, suggesting the presence of selective metal resistance mechanisms. Nature can address heavy metal pollution through microorganisms that adapt to contaminated environments. These microbes develop certain resistance mechanisms and often form biofilms, which help in metal sequestration and remediation by secreting exopolysaccharides (EPS) that bind positively charged metal ions and produce enzymes and biosurfactants [77,78]. While the exact effects of heavy metals on biofilms are not fully understood, some studies have demonstrated the ability of biofilms to remove Cr (VI). For example, biofilms formed by *Pseudomonas putida*, *Arthrobacter viscosus*, or mixed microbes from activated sludge have shown Cr (VI) reduction and immobilization [79]. In our study, although biofilm formation was limited under elevated chromium levels, its presence could enhance the survival of CRB14 in harsh environments with high metal concentrations [57].

According to recent research, microorganisms suitable for bioremediation may promote plant growth. This is attributed to their abilities to detoxify and degrade toxins, as well as their production of growth-promoting metabolites. Our study revealed several plant growth-promoting (PGP) properties of CRB14. These include nitrogen fixation (10.34 μg/mL) and positive for ammonia production, cellulase production, phosphate solubilization (1.05 μg/mL), IAA production (28.165 μg/mL), and siderophore production (49.02 psu). IAA is a plant hormone that stimulates root growth and development. Nitrogen fixation and ammonia production by CRB14 can enrich the soil with nitrogen, a vital nutrient for plant growth. Phosphate solubilization by CRB14 can increase the availability of phosphorus in the soil, another essential plant nutrient. Furthermore, CRB14 demonstrated siderophore production, which can help plants acquire iron, a vital nutrient for growth [80]. These iron acquisition systems might indirectly contribute to Cr (VI) reduction. On the other hand, CRB14 has cellulose degradation ability. Plants typically contain up to 60% cellulose, the decomposition of cellulose is a key activity of soil bacteria and is vital to the energy flow through soils and the cycling of N, P, and S, where immobilization generally accompanies cellulose decomposition [81]. These PGP traits suggest CRB14’s potential as a biofertilizer that can enhance plant growth and metal stress tolerance.

The genetic determinants of resistance to heavy metals are often found in plasmids and transposons [58]. Plasmid analysis revealed no detectable plasmids in CRB14, suggesting chromosomal residence of Cr resistance genes. A similar result is also shown in *Bacillus* spp. [82]. By analyzing PCR amplification products, it was revealed that the Cr-tolerance-related gene ChrA exists in the genome of CRB14. Chr-A encodes transporters involved in the transport of Cr(III) and Cr(VI) through functions such as creating a transmembrane proton gradient, generating membrane potential, and facilitating electron transfer [81]. Upon exposure to chromium stress, Cr(VI) enters bacterial cells via sulfate ion channels and is carried out by ChrA transmembrane proteins, thus mitigating the harmful effects of hexavalent chromium [20,21,22]. Conversely, the absence of the *ycnD* gene, associated with FMN reductase, suggests alternative pathways for Cr (VI) reduction might be present in the genome of CRB14.

In support of the observational findings, the genome of CRB14 was thoroughly analyzed, revealing the presence of chromium and several other heavy metal resistance genes. This provides compelling evidence for the chromosomal location of these resistance genes in the isolate. The whole-genome analysis also uncovered 34 putative genomic islands (GIs) within its genome. GIs play a crucial role in the dissemination of heavy metal resistance genes among bacteria, enhancing their adaptability in metal-contaminated environments [83]. Researchers have shown that these discrete DNA segments, acquired via horizontal gene transfer (HGT), significantly contribute to bacterial genome plasticity by integrating beneficial genetic material in response to environmental pressures [84]. Via HGT, GIs may harbor genes involved in detoxifying or resisting toxic metals, offering a survival advantage in metal-rich environments. In turn, this genetic exchange promotes environmental adaptation and can result in the evolution of highly resistant bacterial strains capable of thriving under extreme conditions.

Functional annotation implies the involvement of diverse metabolic pathways and the potential utilization of siderophores for Cr resistance. Such resistance was observed for cadmium [85]. This knowledge could contribute to the development of bioremediation strategies for chromium-contaminated environments. Different mechanisms of microbial interaction with chromium biosorption and bioreduction have been considered for the development of biotechnological strategies for the removal of Cr from the environment. Biotechnological process of interest is based on the bacterial cells and enzymes immobilized in different polymer matrices such as agar layers, polyhydroxyalkanoate granules, calcium alginate, alginate beads, and poly(vinyl alcohol) alginate, which have proven to be effective for Cr(VI) reduction. The ChromeBac™ system works by immobilizing *Acinetobacter haemolyticus* EF369508, which is then placed on rubber wood sawdust inside a bioreactor. Once 90% of the initial Cr(VI) (30–60 mg L^−1^) was reduced, immobilized chromate reductase alginate beads packed in a flow-through column reduced the remaining Cr(VI) to 1.0–1.5 mg/L [86]. Future research is to be focused on the functional characterization of the identified Cr resistance genes paving the way for its potential application in genetic engineering for bioremediation.

## 5. Conclusions

This study presents the characterization of a chromium-reducing bacterium, *Bacillus tropicus* CRB14, collected from tannery effluent. Both experimental data and genome analysis revealed the presence of several genetic determinants related to resistance against chromium and other heavy metals, including cadmium, cobalt, iron, lead, arsenic, and zinc. Furthermore, CRB14 exhibited strong plant growth-promoting capabilities, such as efficiently solubilizing inorganic phosphate, producing siderophores to enhance nutrient uptake, and synthesizing indole-3-acetic acid to stimulate plant growth. To the best of our knowledge, this is the first report offering new insights into chromium and multi-metal resistance in this species of *Bacillus*. Future research will further elucidate the mechanisms and pathways involved in CRB14’s multi-metal resistance, potentially supporting its application in heavy metal-polluted environmental and agricultural sites. 

## Figures and Tables

**Table 1:** Primers for PCR amplification.

Primer Name	Primer Sequence (5′ to 3′)	Amplicon Size
ChrA F	GCGAAAACGAAATCTGAAGC	268 bp
ChrA R	AAACGGGATGATGACGAAAG	
YcnD F	CCAAAATTGCGCTTGAAGAT	342 bp
YcnD R	TCACGGATGTGCGGATAGTA	

**Table 2:** Genes related to heavy metal tolerance in the CRB14 genome.

Heavy Metals	Gene Name	Product
Cr	ChrA	Chromate transport protein
Co, Zn, Cd	CzcB	Cobalt-zinc-cadmium resistance protein
Cu	CutC	Cytoplasmic copper homeostasis protein
	CopC	Copper resistance protein
	CopD	Copper resistance protein

## Data Availability

The raw data supporting the conclusions of this article will be made available by the authors on request.

## Supplementary Materials

The following supporting information can be downloaded at: https://www.mdpi.com/article/10.3390/microorganisms12122633/s1, Figure S1. Optimization of physical factors (pH, temperature, shaking speed) for the growth of CRB14. After incubation, the bacteria exhibited the best growth at (a) 35 °C with (b) 150 rpm shaking at (c) pH 8; Figure S2. Plant growth promoting (PGP) activities determination of the isolate CRB14; Figure S3. Molecular Characterization of CRB14. (a) Plasmid profiling of CRB14 shows no bands in the gel, indicating the absence of plasmids in the isolate. (b) PCR amplification of Cr(VI) Resistant *chrA* and *ycnD* Gene. Lane 1 and Lane 2 represent the PCR products using *chrA* and *ycnD* gene-specific primers, respectively. A band is visible in Lane 1, confirming the presence of the *chrA* gene, while no band is observed in Lane 2, indicating the absence of the *ycnD* gene in the CRB14 genome; Figure S4. Species identification based on whole-genome sequencing. A heat map illustrating the similarity levels based on the average nucleotide identities (ANI) of the whole genomes of 40 related strains. ANI percentages are visualized with color intensity—red for higher similarity and blue for lower. The two most similar species, *Bacillus tropicus* strain EMB20 and *Bacillus tropicus* CRB14, are highlighted within a box; Table S1. MIC (Minimum inhibitory concentration) determination for all isolates; Table S2. Microtiter Plate Assay (Quantitative Assays for Biofilm Formation); Table S3. General features of the isolate CRB14; Table S4. List of antibiotics and associated resistance mechanism found in CRB14; Table S5. Biosynthetic gene cluster family (BiG-FAM) database with the summary of all best BGC-to-GCF pairings.
