# Supplementary material for: Characterization and Molecular Insights of a Chromium-Reducing Bacterium Bacillus tropicus
Source: Microorganisms. 2024 Dec 19;12(12):2633. doi: 10.3390/microorganisms12122633 (PMC11676387; doi:10.3390/microorganisms12122633)
Supplement: Supplementary file 1 [file microorganisms-12-02633-s001.zip › microorganisms-3229196-supplementary.pdf]

microorganisms

MDPI

Article

Characterization and Molecular Insights of a Chromium-Reducing Bacterium *Bacillus tropicus*

Shanjana Rahman Tuli [1], Md. Firoz Ali [1,2], Tabassum Binte Jamal [1], Md. Abu Sayem Khan [1,3], Nigar Fatima [1], Irfan Ahmed [1], Masuma Khatun [1] and Shamima Akhtar Sharmin [1,*]

Table S1: MIC (Minimum inhibitory concentration) determination for all isolates

Cr (VI) Concentrations (mg/mL)	Isolate ID				
	CRB8	CRB10	CRB12	CRB14	CRB16
1000	+	+	+	+	+
2000	+	+	+	+	+
3000	+	-	+	+	-
4000	-	-	+	+	-
5000	-	-	-	+	-
6000	-	-	-	-	-

'+' indicates growth of isolates and '-' indicates no growth of isolates

Table S2: Microtiter Plate Assay (Quantitative Assays for Biofilm Formation)

Chromium(VI) (mg/mL)	OD_c	OD	Biofilm Formation
25	0.040	0.0527	Weak Biofilm Producer($OD > OD_c$)
50	0.045	0.0537	Weak Biofilm Producer($OD > OD_c$)
100	0.044	0.0560	Weak Biofilm Producer($OD > OD_c$)
200	0.049	0.0529	Weak Biofilm Producer($OD > OD_c$)

Table S3: General features of the isolate CRB14

Attribute	Value	Attribute	Value
Genome size (bp)	52,17,143	Contigs	344
GC content (%)	35.3	Genomic island number	34
CDS	5255	CRISPR repeats	4
tRNA number	37	Cas Genes	4
rRNA number	3	Cas Clusters	2
tmRNA	1	ARGs	14

Table S4: List of antibiotics and associated resistance mechanism found in CRB14

Antibiotics	Drug Class	AMR Gene Family	Resistance Mechanism
vancomycin	glycopeptide	glycopeptide resistance gene cluster	antibiotic target alteration
teicoplanin	glycopeptide	glycopeptide resistance gene cluster	antibiotic target alteration
fosfomycin	phosphonic acid	fosfomycin thiol transferase	antibiotic inactivation
tetracycline	tetracycline	tetracycline-resistant ribosomal protection protein	antibiotic target alteration
doxycycline	tetracycline	tetracycline-resistant ribosomal protection protein	antibiotic target alteration
minocycline	tetracycline	tetracycline-resistant ribosomal protection protein	antibiotic target alteration
chlortetracycline	tetracycline	tetracycline-resistant ribosomal protection protein	antibiotic target alteration
demeclocycline	tetracycline	tetracycline-resistant ribosomal protection protein	antibiotic target alteration
oxytetracycline	tetracycline	tetracycline-resistant ribosomal protection protein	antibiotic target alteration

Table S5: Biosynthetic gene cluster family (BiG-FAM) database with the summary of all best BGC-to-GCF pairings

Class	Completeness	Distance
NRP:metallophore	fragmented	725
Tarpene	complete	130
RiPP:RRE-containing	complete	843
Betalactone	complete	671
RiPP:RiPP-like	complete	763
RiPP:RiPP-like	complete	441
RiPP:lanthipeptide	fragmented	378
RiPP:LAP	complete	635
Siderophore	complete	850

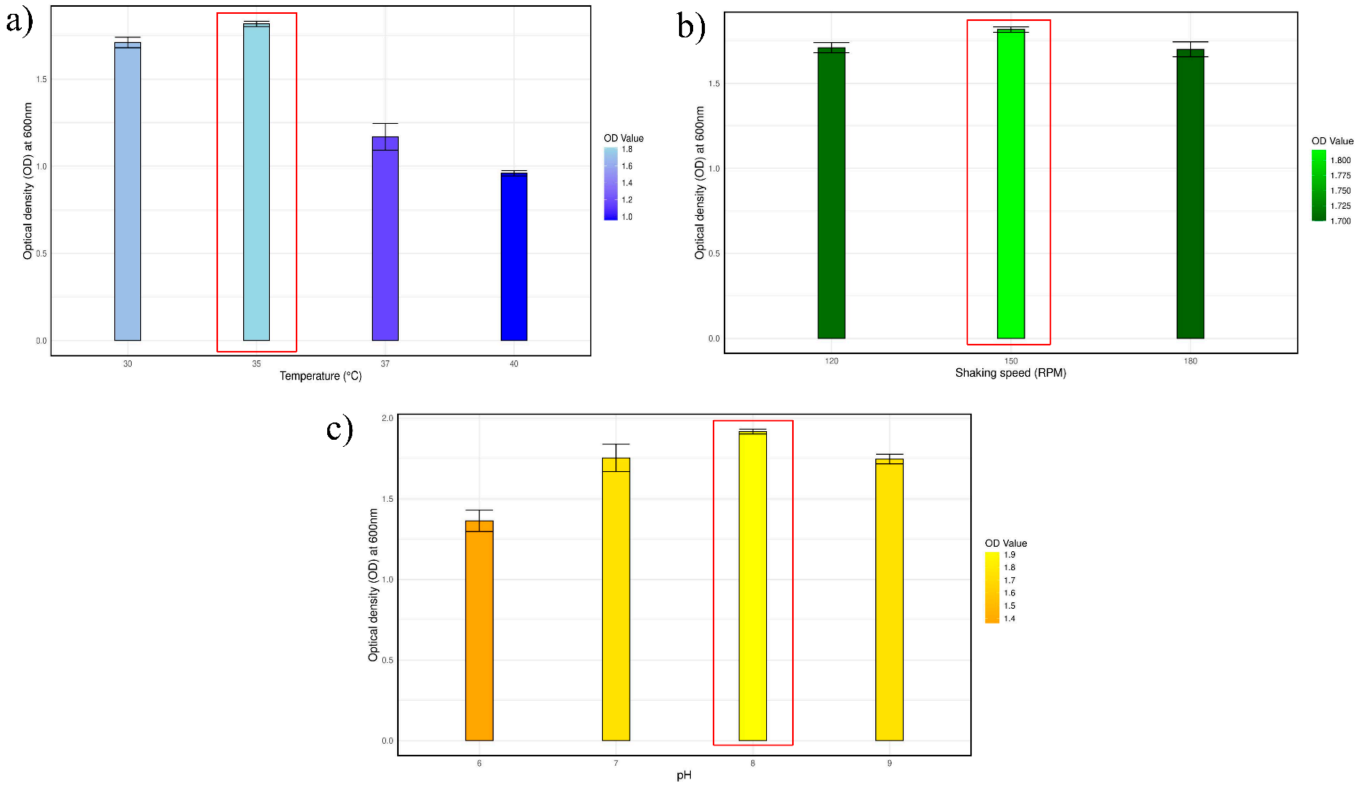

Figure S1. Optimization of physical factors (pH, temperature, shaking speed) for the growth of CRB14. After incubation, the bacteria exhibited the best growth at (a) 35°C with (b) 150 rpm shaking and (c) pH 8.

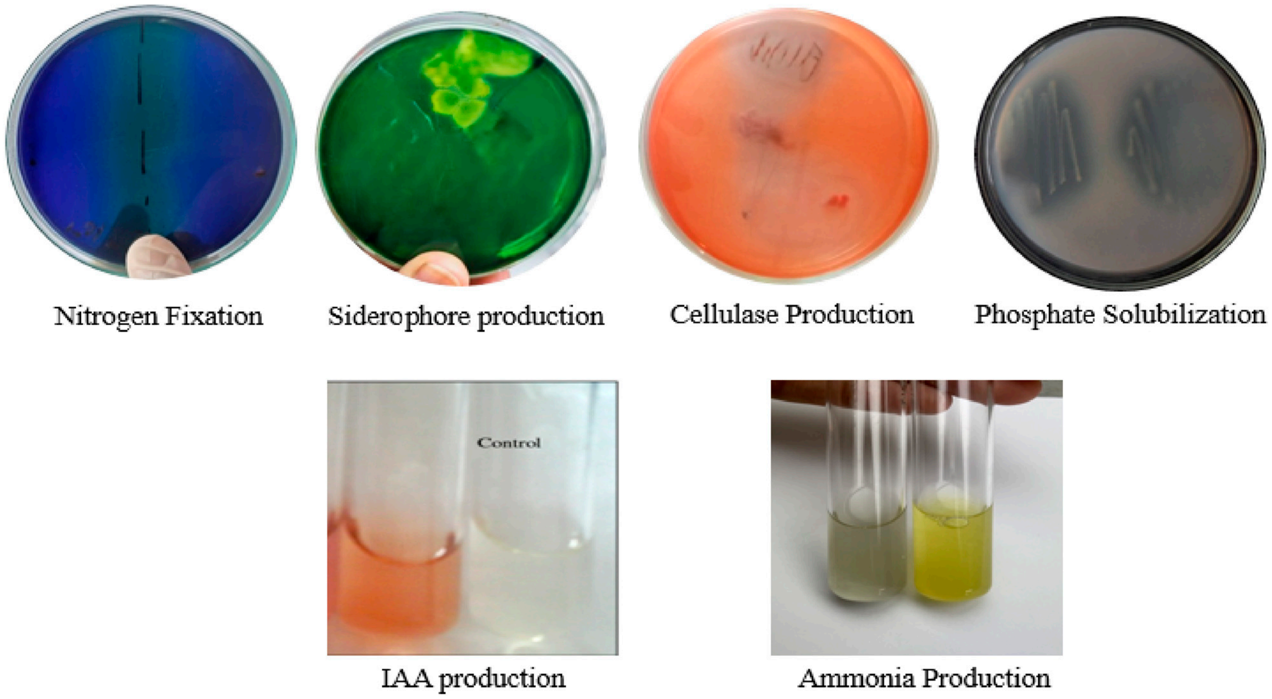

Figure S2. Plant growth promoting (PGP) activities determination of the isolate CRB14.

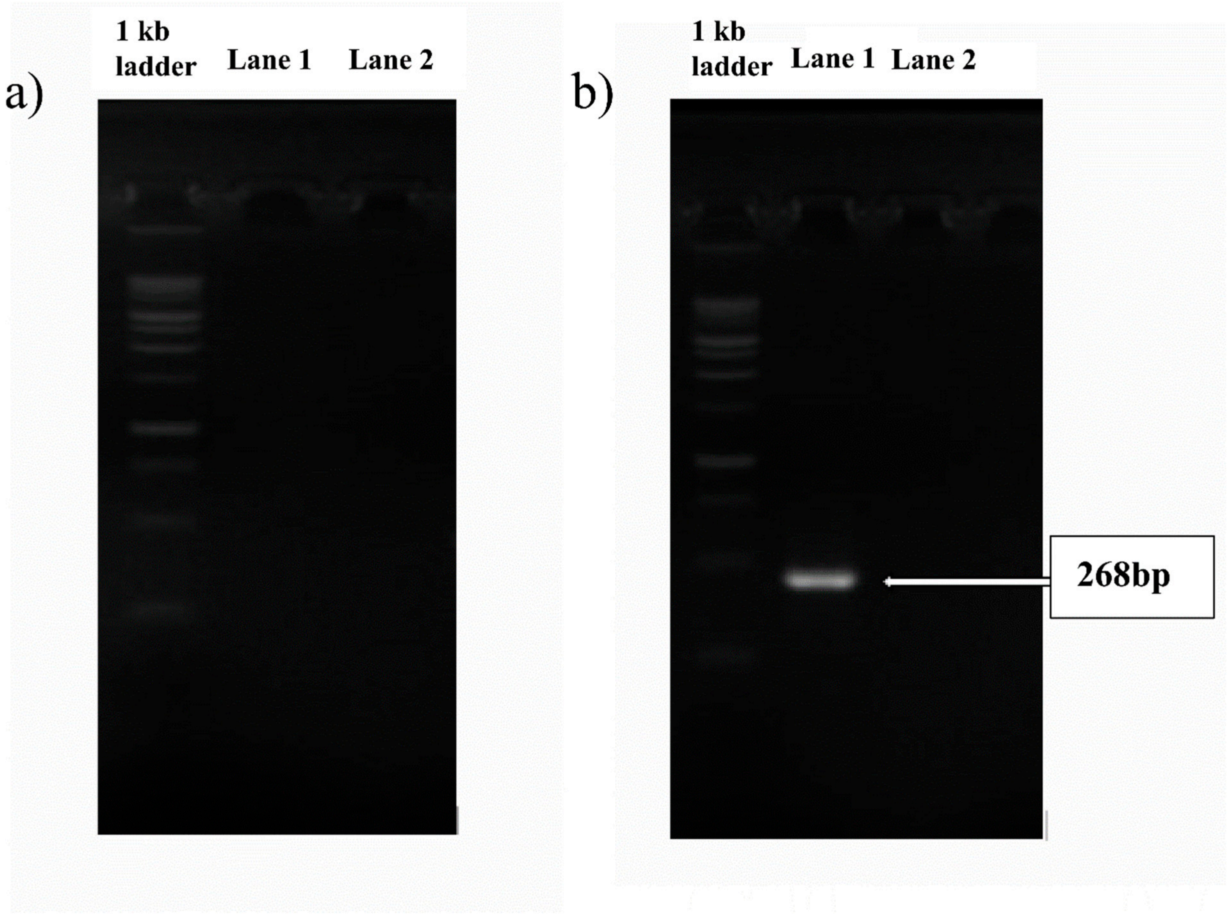

Figure S3. Molecular Characterization of CRB14. a) Plasmid profiling of CRB14 shows no bands in the gel, indicating the absence of plasmids in the isolate. b) PCR amplification of Cr(VI) Resistant chrA and ycnD Gene. Lane 1 and Lane 2 represent the PCR products using chrA and ycnD gene-specific primers, respectively. A band is visible in Lane 1, confirming the presence of the chrA gene, while no band is observed in Lane 2, indicating the absence of the ycnD gene in the CRB14 genome.

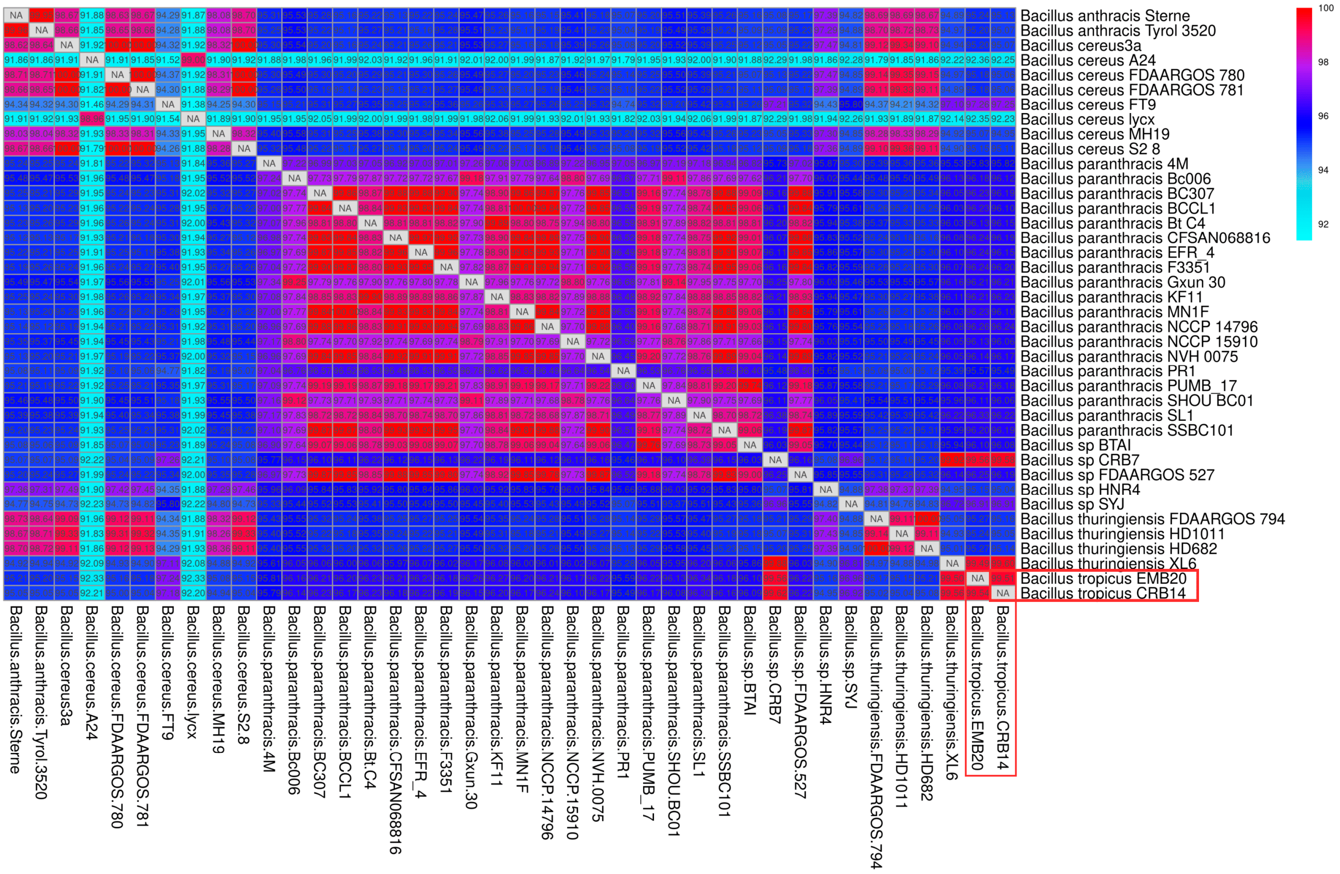

Figure S4. Species identification based on whole-genome sequencing. A heat map illustrating the similarity levels based on the average nucleotide identities (ANI) of the whole genomes of 40 related strains. ANI percentages are visualized with color intensity red for higher similarity and blue for lower. The two most similar species, *Bacillus tropicus* strain EMB20 and *Bacillus tropicus* CRB14, are highlighted within a box.
